# Aberrant salience correlates with psychotic dimensions in outpatients with schizophrenia spectrum disorders

**DOI:** 10.1186/s12991-022-00402-5

**Published:** 2022-07-03

**Authors:** Valentina Pugliese, Renato de Filippis, Matteo Aloi, Paola Rotella, Elvira Anna Carbone, Raffaele Gaetano, Pasquale De Fazio

**Affiliations:** 1 Psychiatry Unit, Department of Health Sciences, University Magna Graecia of Catanzaro, Viale Europa, 88100 Catanzaro, Italy; 2 Department of Mental Health of Catanzaro, Lamezia Terme, Italy

**Keywords:** Aberrant salience, Negative symptoms, Positive symptoms, Postural mannerisms, Social withdrawal, Alogia, Blunted affect, Delusions, Disorganization, Schizophrenia

## Abstract

**Background:**

Aberrant salience is a well-known construct associated with the development and maintenance of psychotic symptoms in schizophrenia. However, only a few studies have investigated aberrance salience as a trait, with no study investigating the association between the five aberrant salience domains and psychotic symptoms. We aimed to explore the role of aberrant salience and its domains on psychotic dimensions in both clinically remitted and non-remitted patients.

**Methods:**

A sample of 102 patients diagnosed with schizophrenia spectrum disorders was divided according to the Positive and Negative Syndrome Scale (PANSS) remission criteria into two groups: remitted and non-remitted. Differences regarding psychotic symptomatology assessed by the PANSS and aberrant salience measured by the Aberrant Salience Inventory (ASI) were explored. Finally, a correlation analysis between the PANSS and the ASI was run.

**Results:**

Significantly higher ASI scores were evident among non-remitted patients. Positive symptoms (i.e. delusions, conceptual disorganization, and hallucinatory behaviour) and general psychopathology (i.e. postural mannerisms, unusual thought content) were correlated to the aberrant salience subscales ‘sharpening of senses’, ‘heightened emotionality’ and ‘heightened cognition’ and with the ASI total score. Significant correlations emerged between negative symptoms (blunted affect and social withdrawal) and ‘heightened cognition'. Finally, lack of spontaneity of conversation was related to the subscales ‘heightened emotionality’ and ‘heightened cognition’, as well as to the ASI total score.

**Conclusions:**

These preliminary results support the hypothesis of an association between aberrant salience and psychotic symptoms in schizophrenia. Further research is needed, especially into the mechanisms underlying salience processing, in addition to social and environmental factors and cognitive variables.

## Background

Aberrant salience is a well-known construct associated with the development and maintenance of psychotic symptoms [1]. Much has been speculated about the origin of delusions; current knowledge considers that delusions are caused and maintained by dysregulated discharges of dopaminergic neurons that force patients to focus their attention to otherwise irrelevant external stimuli, thus attributing a pathological and wrong meaning. This phenomenon is known as ‘aberrant salience attribution’ [2–4]. This aberrant relevance causes a conflict that could be cognitively resolved with the creation of the first delusional thought and, eventually, end in complete delusion with a self-sustaining course [4, 5]. Thus, delusions might represent a cognitive strain to sort out confusing and unintelligible stimuli that make sense of subsequent aberrant salient experiences, whereas hallucinations may reflect a direct experience of aberrant salience [4]. Indeed, according to the original Kapur’s conceptualization, hallucinations are aberrant perceptions belonging to the wide model of psychosis, also defined as exaggerated, amplified, and aberrantly recognized internal percepts. In this framework, hallucinations arise from the internal representations of percepts and memories, with a huge gradation of severity, ranging from stimuli similar to own “internal thoughts” up to clear “voices” coming from without [4].

There is some evidence on the role of aberrant salience in schizophrenia, even if the results are somewhat inconsistent and inconclusive [6]. Data from measures of aberrant salience, such as the Salience Attribution Task (SAT) [7] and the Aberrant Salience Inventory (ASI) [8], which measure implicit (behavioural) and explicit (self-report) aberrant and adaptive salience, respectively, show increased aberrant salience in patients with schizophrenia compared to controls [8, 9]. Additionally, aberrant salience is associated with abnormal beliefs [10], perceptual aberration and magical ideation [8, 11] in individuals with no history of psychosis, as well as in individuals at ultra-high risk (UHR) to develop psychosis and first-episode psychosis (FEP) patients [12–14], suggesting also a link between aberrant salience and the risk of developing psychosis.

Conversely, data from other studies with the SAT suggest that salience is normal in schizophrenia [7, 15] and several studies did not find any direct relationship between the ASI and the SAT [6].

The aberrant salience hypothesis of schizophrenia may also be extended to explain social cognition impairments and negative symptoms [16]. Indeed, findings suggest that the degree of salience coding in the prefrontal cortex could be inversely correlated with anhedonia [17]. Disrupted salience processing, including the salience network and a disconnection of the salience and reward networks, may account for the lack of motivation in patients with schizophrenia [18]; nevertheless, only a few studies have supported the relationship between the salience network and negative symptoms [19].

To date, only one study has evaluated the association between the aberrant salience ‘trait’ and psychotic symptoms, in a mixed sample that also included patients with schizophrenia [20]. However, the authors only found an association between first rank symptoms and aberrant salience, regardless of the diagnosis, and found no correlation between aberrant salience and positive/negative symptoms or general psychopathology assessed by the Positive and Negative Syndrome Scale (PANSS).

To the best of our knowledge, no studies have investigated the association between the five aberrant salience domains—feelings of increased significance, sense sharpening, impending understanding, heightened emotionality and heightened cognition—according to Kapur’s conceptualization [4] and psychotic symptoms in patients affected by schizophrenia spectrum disorders (SSD).

We hypothesize that the aberrant salience trait and its domains are related to more severe general psychopathology as well as positive and negative symptoms. Therefore, the goal of this study was to investigate the relationship between aberrant salience and psychotic symptoms in a sample of SSD patients, according to clinical remission state. In particular, we aimed to study the correlation between each aberrant salience subdomain and psychosis, to better understand their relationships.

## Methods

### Participants and procedures

This cross-sectional study was carried out at the Psychiatry Unit of the University Hospital Mater Domini of Catanzaro (Italy) in accordance with the latest version of the Declaration of Helsinki [21]. The study protocol was approved by the local research ethics committee. All patients signed written informed consent before any data were collected.

A total of 122 outpatients aged 18–65 years were consecutively recruited from August 2019 to August 2020. Following current definition of SSD [22, 23], we included all patients with a diagnosis of schizophrenia or schizoaffective disorder according to Diagnostic and Statistical Manual of Mental Disorders, Fifth Edition (DSM-5) criteria [24], formulated through the Structured Clinical Interview for DSM-5 (SCID-5-CV) [25] by experienced psychiatrists who used these tools in their daily clinical practice, after a minimum follow-up period of 12 months in our unit. The daily dose of prescribed antipsychotic drugs was expressed as the equivalent daily dose of chlorpromazine, based on the international consensus study [26].

We settled on the following exclusion criteria: (1) patients with schizotypal personality disorder, brief limited intermittent psychotic symptoms (BLIPS), delusional disorder, or acute psychotic episode (according to clinical evaluation); (2) patients diagnosed with dementia, intellectual disability or other severe medical conditions associated with psychiatric symptoms potentially biasing assessment; (3) patients with active substance abuse in the last 6 months; (4) patients affected by conditions that did not allow them to complete the assessment, such as language problems, dyslexia or lack of knowledge of Italian language; and (5) those who have not provided valid informed consent.

Of the 122 patients initially enrolled in the study, 20 were excluded. Seven patients (5.7%) dropped out before the end of the assessment and thus were excluded from the study, and 13 (10.7%) were not eligible for various reasons: five patients were not interested in participating and eight met the exclusion criteria (i.e. intellectual disability, active substance use). Thus, the final sample was made up of 102 participants (dropout rate 16.4%) who completed the study, including:

• 78 patients diagnosed with schizophrenia
• 24 patients diagnosed with schizoaffective disorder

Then, the sample was divided into two groups according to the current clinical remission state as defined by the PANSS remission criteria elaborated by Andreasen et al. [27] and set up by the Remission in Schizophrenia Working Group [28]*.* The eight items considered include delusions (P1), conceptual disorganization (P2), hallucinatory behaviour (P3), blunted affect (N1), social withdrawal passive/apathetic (N4), lack of spontaneity of conversation (N6), mannerisms and posturing (G5) and unusual content of thought (G9); symptomatic remission is defined as a score of 3 (‘mild’) or lower, on all items considered.

### Measures

#### Clinical and sociodemographic characteristics

An ad hoc schedule was administered in order to assess the demographic and clinical characteristics of the sample. Collected data included general medical information, psychiatric familial and personal history, age at onset of illness, duration of untreated psychosis (DUP), previous psychiatric hospitalizations and antipsychotic therapy.

#### Assessment

The *Aberrant Salience Inventory (ASI)* [8] is a self-administered test, consisting of 29 items with a dichotomous yes/no response, that assesses individual aberrant salience and psychotic proneness. The scale investigates the presence of five factors correlated with each other and in line with Kapur’s conceptualization [4]: feelings of increased significance (7 items), sense sharpening (5 items), impending understanding (5 items), heightened emotionality (6 items) and heightened cognition (6 items). The Italian version of the ASI used in this study confirmed the good psychometric properties of the scale, with gold standard values of internal coherence and stability at retest [29].

The *Positive and Negative Symptom Scale (PANSS)* [28] is a standardized clinical interview that rates the presence and severity of positive and negative symptoms, as well as general psychopathology, for patients with schizophrenia within the past week. The instrument consists of 30 items evaluated on a seven-point Likert scale (1 = absent to 7 = extreme): the positive and negative subscales consist of seven items each (score: 7–49), whereas the general psychopathology subscale consists of 16 items (score: 16–122).

In this study, with the aim of considering the sample based on remission state [30], we applied the eight items of the PANSS remission criteria: delusions (P1), conceptual disorganization (P2), hallucinatory behaviour (P3), blunted affect (N1), social withdrawal passive/apathetic (N4), lack of spontaneity of conversation (N6), mannerisms and posturing (G5) and unusual content of thought (G9).

### Statistical analyses

Data were analysed using the Social Sciences Statistical Package, Version 26.0 (SPSS Inc., Chicago, IL, USA). In order to select appropriate methods of statistical analysis, the normal distribution was checked using the Shapiro–Wilk test.

The variables of the PANSS and ASI were not normally distributed; therefore, the Mann–Whitney *U* test was used for group comparison. Additionally, effect size *r* was calculated for significant results. Values of 0.2, 0.5 and > 0.8 can be categorized into small, moderate and large effect size, respectively [31].

Pearson’s correlation was used to assess the correlation between psychotic symptomatology (PANSS) and aberrant salience (ASI). The statistical significance level was set at *p* < 0.05.

## Results

Table 1 summarizes the demographic and clinical characteristics of the sample. Most patients (77%) reported no familial history of SSD. The age of onset of the disorder was, on average, 23.2 ± 6.3 years, while the mean DUP was 1.6 ± 2.1 years. The average dose of antipsychotics was 467.7 ± 185.6 mg/day (chlorpromazine equivalents). Almost 60% of patients were not clinically remitted.

**Table 1:** Socio-demographic and clinical characteristics

		*N* = 102	
		*Fr*	*%*
Mean age (years)^a^		38.6	(10.2)
Gender	Male	65	(63.7)
	Female	37	(36.3)
Civil status	Married	13	(12.7)
	Divorced	8	(7.8)
	Single	81	(79.4)
Education (years)^a^		12.9	(3.3)
Employment	Employed	21	(20.6)
	Unemployed	52	(51.0)
	On pension	14	(13.7)
	Unpaid activity	6	(5.9)
	Invalid/retired	9	(8.8)
Living status	Alone	9	(8.8)
	Parents	56	(54.9)
	Partner	13	(12.7)
	Other	24	(23.5)
Diagnosis	Schizophrenia	78	(76.5)
	Schizoaffective disorder	24	(23.5)
Familiarity for psychosis	Yes	25	(24.5)
	No	77	(77.5)
Age at onset (years)^a^		23.2	(6.3)
Duration of untreated psychosis (years)^a^		1.6	(2.1)
Previous psychiatric hospitalizations^a^		2.3	(3.0)
Daily equivalent dose of chlorpromazine (mg)		467.7	(185.6)
Remission*	Yes	43	(42.2)
	No	59	(57.8)

^a^Data are expressed as means and (SD)

^*^Symptomatic remission is defined as a score of 3 (“mild”) or less on all of eight items (P1, P2, P3, N1,N4, N6, G5, G9) at PANSS

Patients who were not in remission scored significantly higher than remitted patients on the ASI subscales ‘sharpening of senses’ (SS: *p* = 0.002), ‘heightened emotionality’ (HE; *p* = 0.036), ‘heightened cognition’ (HC: *p* = 0.007) and the total score (*p* = 0.010), with an ES that ranged from small to moderate (0.21–0.63) (Table 2).

**Table 2:** Comparison of scores obtained at PANSS and ASI according to the remission

		Non-remitter	Remitter	*U*	*z*	*p*	*r*^*a*^
		*N* = 59	N = 43				
		Median	Median				
PANSS	Delusions (P1)	4.0	2.0	370.0	− 6.271	** < 0.001**	**0.63**
	Conceptual disorganization (P2)	4.0	3.0	464.0	− 5.805	** < 0.001**	**0.54**
	Hallucinatory behaviour (P3)	3.0	1.0	601.0	− 4.694	** < 0.001**	**0.46**
	Blunted affect (N1)	4.0	3.0	436.5	− 6.060	** < 0.001**	**0.56**
	Social withdrawal passive/apathetic (N4)	4.0	3.0	466.5	− 5.625	** < 0.001**	**0.55**
	Lack of spontaneity of conversation (N6)	4.0	2.0	663.5	− 4.231	** < 0.001**	**0.41**
	Mannerisms and posturing (G5)	3.0	2.0	685.0	− 4.140	** < 0.001**	**0.42**
	Unusual thought content (G9)	3.0	3.0	411.0	− 6.038	** < 0.001**	**0.56**
ASI	Increased significance	5.0	4.0	1048.5	− 1.514	0.130	
	Sharpening of senses	3.0	2.0	824.0	− 3.066	**0.002**	**0.30**
	Impending understanding	3.0	2.0	1041.5	− 1.564	0.118	
	Heightened emotionality	4.0	3.0	963.5	− 2.093	**0.036**	**0.21**
	Heightened cognition	4.0	3.0	874.0	− 2.710	**0.007**	**0.26**
	Total ASI	20.0	14.0	889.5	− 2.572	**0.010**	**0.25**

Symptomatic remission is defined as a score of 3 (“mild”) or less on all of eight items (P1, P2, P3, N1, N4, N6, G5, G9)

^a^Only effect sizes of significant differences are displayed

*PANSS* positive and negative schizophrenic symptoms, *ASI* aberrant salience inventory

Significant results are in bold characters

Table 3 shows the correlation between psychotic symptoms (PANSS) and aberrant salience (ASI). Positive symptomatology (P1, P2, P3) and general psychopathology (G5, G9) were correlated with the subscales ‘sharpening of senses’, ‘heightened emotionality’ and ‘heightened cognition’, and with the total ASI score. Regarding negative symptomatology, we found a significant correlation between N1 and N4 and the subscale ‘heightened cognition’; N6 was related to the subscales ‘heightened emotionality’ and ‘heightened cognition’, as well as with the total ASI score.

**Table 3:** Correlations between psychotic symptoms and ASI subscales

	SS	HE	HC	ASI	P1	P2	P3	N1	N4	N6	G5	G9
SS	–											
HE	0.570^**^	–										
HC	0.704^**^	0.653^**^	–									
ASI	0.803^**^	0.839^**^	0.867^**^	–								
P1	0.444^**^	0.309**	0.399^**^	0.396^**^	–							
P2	0.308^**^	0.236^*^	0.262^**^	0.273^**^	0.577^**^	–						
P3	0.237^*^	0.223^*^	0.265^**^	0.243^*^	0.644^**^	0.498^**^	–					
N1	0.135	0.187	0.244^*^	0.163	0.381^**^	0.485^**^	0.423^**^	–				
N4	0.125	0.156	0.222^*^	0.131	0.404^**^	0.508^**^	0.445^**^	0.777^**^	–			
N6	0.149	0.233^*^	0.216^*^	0.200^*^	0.276^**^	0.383^**^	0.369^**^	0.696^**^	0.642^**^	–		
G5	0.201^*^	0.219^*^	0.309^**^	0.255^**^	0.413^**^	0.530^**^	0.370^**^	0.500^**^	0.478^**^	0.505^**^	–	
G9	0.270^**^	0.255^**^	0.319^**^	0.278^**^	0.795^**^	0.482^**^	0.650^**^	0.428^**^	0.456^**^	0.427^**^	0.404^**^	–

*SS* sharpening of senses, *HE* heightened emotionality, *HC* heightened cognition, *ASI* Aberrant Salience Inventory total score, *P1* delusions, *P2* conceptual disorganization, *P3* hallucinatory behavior, *N1* blunted affect, *N4* social withdrawal passive/apathetic, *N6* lack of spontaneity of conversation, *G5* mannerisms and posturing, *G9* unusual thought content

^*^p < 0.05

^**^p < 0.01

Significant results are in bold characters

## Discussion

The aim of this study was to assess the association between the aberrant salience trait and the severity of psychotic symptoms. The results of data analysis showed not only significantly higher ASI scores among non-remitted patients, but also an association between aberrant salience and positive symptoms (i.e. delusions, conceptual disorganization, hallucinatory behaviour) and general psychopathology (i.e. postural mannerisms, unusual thought content), as well as with negative symptoms (i.e. blunted affect, social withdrawal, lack of spontaneity of conversation).

This result is consistent with previous research that assessed the interplay of momentary aberrant salience in the development of paranoid [32] and psychotic experiences [33] in patients with SSD.

However, to our knowledge, this was the first study to examine the association between psychotic symptoms and aberrant salience domains in patients with SSD.

Dopaminergic dysfunction may contribute to a misattribution of salience involving both rewarding and aversive signalling; this could lead to the world seeming loaded with significance, generating feelings of apprehension and a sense that the world has changed in some, as yet, uncertain way [34].

According to this model of impaired processing capability, the dysfunction of the attribution of meaning leads to a “defective filter” where dopamine mediates a flood of stimuli together with aberrant significance from a neutral information into a positive or noxious entity [4, 35].

These experiences are characteristic of the prodromal phase of schizophrenia [34]. From this perspective, primary delusions can derive from the prodromal state, such as the individual's explanation of the experience of aberrant salience [36]. Moreover, it is possible that social environmental factors interact with the neurocognitive processes involved in the early stage of psychotic symptoms [37]. In fact, in our previous study, we found that emotional abuse during childhood can be associated with a higher level of aberrant salience in SSD patients [38].

The measurement of aberrant salience as a lifetime condition (trait) and as an episodic element (state) is essential for both a dimensional understanding of psychosis and the management of therapy [32].

In this regard, we hypothesize that aberrant salience differentiation could be explained by different variables, including psychosis severity and phase [10], medication confounding bias [7], or still to the different assessment sensibility [6]. Indeed, the SAT has shown good construct and concurrent validity when used in non-psychotic populations [9, 39], while the ASI better identifies psychotic-like experiences, such as magical ideation, regardless of the diagnosis, discriminating schizophrenia from other major disorders, such as bipolar disorder [8]. Therefore, some authors have speculated that the ASI may be able to identify the aberrant salience as a trait [40], and this could be of particular interest especially when studying non-psychotic populations.

In line with previous findings, we found stronger correlations between aberrant salience and positive symptoms than with negative symptoms [11, 41]. Kapur [4] proposed that antipsychotic drugs, which block the D_2_ dopamine receptors, can reduce positive symptoms by mitigating aberrant motivational salience. A consequence of this hypothesis is that antipsychotic drugs will essentially also reduce adaptive motivational salience, that is, the correct allocation of salience. Unfortunately, this can result both in remission of positive symptoms and increasing negative symptoms as a side effect, mainly related to loss of motivation, such as apathy and anhedonia [4].

Previous research found that schizophrenia patients with florid delusions exhibited significantly more momentary aberrant salience than those without [7]. However, there is a positive relationship between explicit aberrant salience and delusion-like symptoms in people at ultra-high risk of psychosis [10]. Also, a significant association between cannabis-induced psychotic symptom severity (accounting for 37% of the variance in psychotic symptom severity) and aberrant salience processing has been found [42].

On the other hand, to date, the correlation between aberrant salience and negative symptoms has been less investigated. In our study, a relationship emerged between aberrant salience and both expressive deficits (i.e. blunted affect, alogia) and the motivational domain (i.e. asociality).

Motivation impairment, underlying avolition, asociality and potentially anhedonia can derive from different dopamine pathophysiological mechanisms involving the salience system [43]. Indeed, several imaging studies found an association between alterations of the dopamine-dependent response of the ventral striatum to reward anticipation and negative symptoms in patients with schizophrenia [44]. However, in other studies the same alterations were not found to be correlated with negative symptoms [45], thus maintaining uncertainty on the subject.

Schizophrenia is characterized by well-known deficits in the comprehension and production of speech, as well as impairments in the perception and production of gestures [46]. A functional network connectivity analysis conducted on patients with schizophrenia found disrupted communication between the anterior default mode network and the salience network that was positively associated with the severity of blunted affect [47]. In this regard, impaired processing of emotional salience has been proposed as a primary antecedent to the development of psychosis [48] and may be affected according to illness stage [49].

In our study, we found a significant correlation between the subscale ‘heightened cognition’ and positive symptomatology (i.e. delusions, conceptual disorganization, hallucinatory behaviour), postural mannerisms, unusual thought content and both expressive deficits and asociality. Heightened cognition denotes experiences in which individuals feel as if they are part of something important that may not be readily apparent. These perceptual experiences are often accompanied by the feeling that some important understanding may be forthcoming, as a revelation or a sensation of unknown and uncontrolled changes [8]. The presence of elevated levels of heightened cognition was found to be positively correlated to unusual thought content [50] and studies have shown that this dimension of aberrant salience is able to distinguish patients with psychosis from other psychiatric patients, suggesting it may be the most specific for psychosis [51]. Speaking about heightened cognition, the role of neurocognition and intelligence quotient (IQ) prognosis of severe psychiatric diseases is still far from being completely understood [52]. Indeed, there are some emerging data about the correlation between higher IQ and a different prognosis in severe psychiatric conditions, which deserves more future research [53, 54].

Moreover, the subscale ‘heightened emotionality’ was associated with positive symptomatology, postural mannerisms, unusual thought content and alogia. Heightened emotionality represents increased levels of anxiety during the early stages of a psychotic episode in which an individual is trying to make sense of the increased importance of stimuli [4, 8]. Significantly, this dimension is one of the components identified by classical European psychopathology as determining the transition from a pre-delirious state to fully articulated delirious experiences [55].

Finally, the subscale ‘sharpening of senses’ correlated with positive symptomatology, postural mannerisms and unusual thought content. This dimension of aberrant salience involves anomalies of perception in the form of subjective feelings of sharpening of the senses. The experience of aberrant salience may cause a subjective feeling of the senses sharpening as previously non-salient stimuli become salient [4, 8].

Although the preliminary results presented in this study pave the way for a new conceptualization of salience as a trait, and not just as a trigger for psychosis, these findings should be considered in light of several limitations. Firstly, the assessment was conducted through a self-report tool and we could not cross-test aberrant salience with convergent clinician-related tasks (e.g. the SAT) [7]. On the other hand, self-report measures allow the enrolment of large samples; in addition, at present, the ASI is the only available test to measure the aberrant salience trait [8, 56] and it has shown high reliability among patients with a history of psychosis [8]. ASI scores correlate with psychotic-like experiences and discriminate schizophrenia from other psychopathologies, such as bipolar disorder [8], while the SAT shows good construct validity in unaffected individuals [39]. Second, we must acknowledge that our sample included patients taking antipsychotic treatments. While this reflects the naturalistic design of the study, it can clearly affect the relationships between aberrant salience and positive symptoms, considering the antipsychotic medications block the underlying aberrant dopaminergic drive, and given the critical role of dopamine in salience, with a consequential mitigation of the aberrantly salient ideas and perceptions [4]. Therefore, our results must be replicated in a drug-naive sample, preferably at the onset of the disorder, to mitigate this bias. Finally, the association that we found between aberrant salience and positive symptoms may be potentially influenced by their conceptual overlapping. Indeed, we should consider that aberrant salience and psychosis (i.e. experience of delusions, hallucinations, and the secondary related behaviour) are relatively close concepts, even difficult to clinically discriminate from each other. Therefore, although ASI and PANSS are two well-established and solid tools to evaluate aberrant salience and psychosis, this limitation should be considered.

## Conclusions

Our preliminary results seem to support the hypothesis of a dopaminergic regulation of salience that is essential to the pathogenesis of SSD [34]. Indeed, a salience network dysfunction may also play a critical role in explaining the positive, negative and cognitive symptoms of SSD [57].

We found a correlation between the aberrant salience trait and psychotic symptoms. At the beginning of the twentieth century the study of phenomenology seemed destined to never link to the neurobiology, as if they were two irreconcilable aspects of psychiatry. Instead, the application of a more recent dimensional research approach, which aimed to understand salience alterations, allowed to relate neurobiological phenomena (such as dopaminergic alterations) to psychopathological experiences (such as delusions and other psychotic symptoms), thus overcoming this gap [36]. The measurement of aberrant salience could enrich the characterization of genesis and the maintenance of psychotic experiences in patients with schizophrenia. However, the mechanisms underlying salience processing remain unclear and further consideration of social factors and cognitive variables is recommended for future studies.

## Data Availability

Data are available on request due to privacy restrictions.

## Abbreviations

ASI: Aberrant salience inventory
BLIPS: Brief limited intermittent psychotic symptoms
DSM-5: Diagnostic and statistical manual of mental disorders, fifth edition
DUP: Duration of untreated psychosis
FEP: First-episode psychosis
IQ: Intelligence quotient
PANSS: Positive and Negative Symptom Scale
SAT: Salience Attribution Task
SCID-5-CV: Structured Clinical Interview for DSM-5
SPSS: Social sciences statistical package
SSD: Schizophrenia spectrum disorders
UHR: Ultra-high risk
